# Efficacy of erector spine plane block in two different approaches to lumbar spinal fusion surgery: a retrospective pilot study

**DOI:** 10.3389/fmed.2024.1330446

**Published:** 2024-02-14

**Authors:** Jānis Verners Birnbaums, Agnese Ozoliņa, Leonids Solovjovs, Zane Glāzniece-Kagane, Jānis Nemme, Ināra Logina

**Affiliations:** ^1^Riga Stradins University, Riga, Latvia; ^2^Riga East University Hospital, Riga, Latvia; ^3^Daugavpils Regional Hospital, Daugavpils, Latvia; ^4^Orto Clinic, Riga, Latvia

**Keywords:** ESPB, erector spine plane block, regional anesthesia, postoperative pain, lumbar spinal fusion surgery, pain, ultrasound

## Abstract

**Background:**

Erector spine plane block (ESPB) has been widely used in spinal surgery, although there are variable data about its efficacy.

**Objectives:**

This study aimed to evaluate the efficacy of ESPB in elective lumbar spinal fusion surgery patients with two different surgical approaches.

**Materials and methods:**

Retrospectively, 45 elective lumbar transpedicular fusion (TPF) surgery patients undergoing open surgery with different approaches [posterior transforaminal fusion approach (TLIF) or combined posterior and anterior approach (TLIF+ALIF)] were divided into 2 groups: general anesthesia (GA, *n* = 24) and general anesthesia combined with ESPB (GA + ESPB, *n* = 21). The primary outcome was to analyze the efficacy of ESPB in two different surgical approaches in terms of pain intensity in the first 48 h. Secondary: Fentanyl-free patients and opioid consumption in the first 24 h postoperatively. Comparative analysis was performed (SPSS^®^ v. 28.0) (*p* < 0.05).

**Results:**

Out of 45 patients (27 female), 21 received GA + ESPB and 24 received GA. The average age was 60.3 ± 14.3 years. Chronic back pain before the operation was registered in 56% of patients. ESPB was performed in 17 TLIF and in 4 TLIF+ALIF patients. ESPB significantly reduced pain intensity at rest in both surgical approaches 48 h after surgery (*p* < 0.05). The need for postoperative fentanyl infusion was significantly lower in the group treated with GA + ESPB in both surgical approaches than in those who only received GA (29% vs. 77% in TLIF and 0% vs. 80% in TLIF+ALIF); *p* = 0.01 and *p* = 0.004. Additionally, we observed that ESPB provides a good analgesic effect for up to 6.8 ± 3.2 h in the TLIF and 8.9 ± 7.6 h in the TLIF+ALIF approaches. Consequently, ESPB reduced the initiation of the fentanyl compared to GA alone, with a mean difference of 3.2 ± 4.2 h in the TLIF subgroup (*p* = 0.045) and 6.7 ± 5.3 h in TLIF +ALIF (*p* = 0.028). Only in the TLIF+ALIF approach, ESPB reduced the total fentanyl consumption compared to those with GA (1.43 ± 0.45 mg/24 h vs. 0.93 ± 0.68 mg/24 h; *p* = 0.015).

**Conclusion:**

ESPB significantly reduced pain at rest after surgery, the number of patients requiring immediate postoperative fentanyl analgesia, and total fentanyl consumption in both surgical approaches, particularly in TLIF+ALIF. However, the application of ESPB does not always provide completely sufficient analgesia.

## 1 Introduction

Since the human lifespan is rapidly increasing, there is also an increase in the number of patients with degenerative lumbar spondylosis, which is a cause of chronic back pain in up to 80% of cases (1). Nowadays, surgical interventions are gaining in popularity—spinal fusion operations in the US have increased by 77% in the period from 2002 to 2011 (2), and in the UK, the number of surgeries has increased by 63% from 2005 to 2015 (3).

The main indications for spinal fusion surgery are spinal stenosis, spondylolisthesis, and vertebral instability (4). After surgery, most severe pain is expected in the first 3–5 days postoperatively, with a tendency to progress into chronic pain (1, 5–7). Many spinal surgery patients suffer from chronic pain, depression, and restrictions on physical activities (8). Therefore, appropriate postoperative analgesia, with a reduction in opioid consumption, is of superior importance (9, 10).

Currently, the practice of anesthesiology is focused on opioid-sparing postoperative analgesia to avoid opioid-related side effects (2). Peripheral blocks, including erector spine plane block (ESPB), are essential components of multimodal analgesia, which helps to alleviate pain and increase the patient’s comfort (11). It has been widely used in spinal surgery, although there are variable data about its efficacy regarding different surgical approaches, duration of action, and impact on early rehabilitation (12). Recently, a large meta-analysis demonstrated that ESPB used in lumbar spinal surgery was effective in relieving postoperative pain and decreasing the perioperative consumption of opioids (13).

Still, it would be important to understand the impact of the ESPB on pain intensity and opioid consumption after spinal fusion surgeries using two surgical approaches: TLIF and TLIF+ALIF.

In our study, the aim was to look through our first clinical experience with and without ESPB for postoperative analgesia in TPF surgery patients. The primary outcome was to analyze the efficacy of ESPB on pain intensity in the first 48 h for lumbar spinal fusion surgeries with two different surgical approaches. The secondary outcomes were opioid consumption in the first 24 h postoperatively, and the number of fentanyl-free patients was evaluated.

## 2 Materials and methods

### 2.1 Study subjects

This is a retrospective cohort study including 45 adult patients who underwent elective lumbar spinal fusion surgery in the Orto Clinic, Riga, Latvia, from 1 November 2019 to 30 April 2022. All spinal fusion surgeries were performed using two surgical approaches: either posterior transforaminal fusion (TLIF) surgery or combined surgery with posterior and anterior (TLIF+ALIF) approaches. The TLIF approach was performed on multiple surgery levels, but the ALIF approach was performed only on the L5-S1 level.

The inclusion criteria were 8 years of age or older, an ASA score of I–III, and elective lumbar spinal fusion surgery under general anesthesia. The exclusion criteria were known allergic reactions to local anesthetics, signs of local or general infection, pregnancy, history of mental disorders, and failed regional block (immediately reported pain intensity NRS > 6 after surgery).

All the ESPBs were performed by the same anesthesiologist for all included patients starting in September 2021, when ESPBs were introduced in the daily practice for TPF lumbar spinal surgeries. Until then, all patients underwent standardized general anesthesia (GA) without ESPB. Consequently, all patients retrospectively were allocated into two groups: the general anesthesia group (GA, *N* = 24) and GA combined with ESPB (GA + ESPB, *N* = 21). Of those who received GA, 13 underwent the TLIF approach, and 11 had the TLIF + ALIF approach. From those, who received GA + ESPB, 17 underwent TLIF, and only 4 underwent the TLIF + ALIF approach. The patient sample size was based on retrospectively available data, and the incidence of the TPF surgery approach was based on surgical indications; therefore, the sample size in the TLIF+ALIF approach receiving GA + ESPB was lower compared to other groups.

### 2.2 Perioperative care

All patients, with or without ESPB block, received the same standardized GA. It included a premedication of 7.5 mg of oral Midazolam (Dormicum^®^, F. Hoffman-La Roche AG, Switzerland) for 30 min before transfer to the operating room. Induction of GA was provided with midazolam (Dormicum^®^ 5 mg/mL, F. Hoffmann-La Roche Ltd., Switzerland) 2.5 mg, fentanyl (Fentanyl-Kalceks^®^ 0.05 mg/mL, A/S Kalceks, Latvia) 1.5–2 μg/kg, propofol (Propofol^®^ 10 mg/mL, Fresenius Kabi AG, Germany) 2 mg/kg, and cisatracurium (Nimbex^®^, 2 mg/mL, Aspen Pharma Ltd., Ireland) 0.2 μg/kg. Then the patient was intubated. Anesthesia was maintained with sevoflurane (Sevorane^®^, AbbVie S.r.l., Italy) MAC 0.8–1.2, intravenous fentanyl (Fentanyl-Kalceks^®^ 0.05 mg/mL, A/S Kalceks, Latvia) infusion 0.5–1.5 μg/kg/h, and cisatracurium infusion 1–2 μg/kg/min.

For those who received ESPB, after the induction of GA, the patient was intubated and placed in the prone position. Bilateral ultrasound-guided ESPB at the lumbar (L2–L4) level was then performed depending on the spinal fusion level. A high-frequency linear ultrasound transducer was placed in a parasagittal orientation 3 cm laterally from the spinous process. At the spinal lumbar level, the only muscle identified superficial to the hyperdense transverse process is the erector spinae muscle. A 50 mm 22 G ultrasound needle (BRAUN^®^, Germany) was inserted in-plane in a cephalad-to-caudal direction until bone contact with the top of the transverse process. After slight retraction of the needle, 30 mL of 0.35% bupivacaine (Bupivacaine-Grindex, 5 mg/mL, Grindex, Latvia) with 200 mcg epinephrine (Adrenaline, 1 mg/mL, Sopharma Ad. Bulgaria) was injected between the transverse process and erector spinae, observing the cephalad to caudal spread of the local anesthetic. The same procedure was repeated on the contralateral side. During surgery, standard monitoring was performed according to the American Society of Anesthesiology standards.

Postoperatively, hemodynamic monitoring was followed regularly. Fluid management and oxygen supply were provided in the postoperative observational surgical unit for the first 24 h. The patient was assessed for pain control at 0, 1, 6, 12, 24, and 48 h after the surgery using the numeric pain rating scale (NRS). According to the local hospital guidelines, intravenous multimodal analgesia was provided with dexketoprofenum (Dolmen^®^, Berlin-Chemie/Menarini, Germany) 50 mg every 12 h, acetaminophen (Paracetamol, B. Braun Melsungen AG, Germany) 1 g every 6 h, and pregabalin orally (Lyrica^®^, Pfizer, United States) 150 mg every 24 h. For pain exacerbation, if NRS > 6, a fentanyl infusion of 2 mg/50 mL intravenously was started with a rate of 0.5–1 μg/kg/h depending on the response to analgesia. Afterward, total fentanyl consumption was calculated in the first 24 h after surgery. Thromboprophylaxis was provided with enoxaparin 40 mg (Clexane^®^, Sanofi-Aventis S.A. Spain) once daily from the first postoperative day.

### 2.3 Statistical analysis

Statistical analysis was performed using SPSS 26.0 (*Statistical Package for Social Sciences*). The Kolmogorov–Smirnov test was used to evaluate whether datasets conformed to a normal distribution. Continuous variables were presented as mean ± standard deviation (SD), and categorical variables were presented as median ± IQR. Differences in data distribution between the groups were evaluated using a Mann–Whitney U-test for non-parametric datasets and a two-sample *t*-test or ANOVA for datasets conforming with normal distribution. A chi-square test was used for sets of nominal variables. Statistical significance was assumed if the two-tailed *p* < 0.05.

## 3 Results

### 3.1 Clinical course

In total, 45 patients 18 (40%) men and 27 (60%) women were included. The mean age was 60.3 ± 14.3 years. All patients were scheduled for elective lumbar spinal fusion surgery. Of those, 30 patients underwent TLIF, of whom 17 received GA + ESPB and 13 received GA. TLIF+ALIF was performed in 15 patients, of whom 4 received GA + ESPB and 11 received GA. In total, 21 (47%) patients received GA + ESPB, and 24 (53%) were included in the GA group. As shown in Table 1, patients undergoing TLIF+ALIF with GA had a higher body mass index (BMI) compared to those receiving GA + ESPB; *p* = 0.04. Analyzed comorbidities and ASA class were similarly distributed between patients with the two lumbar spinal fusion surgical approaches. Chronic pain (> 3 months) was identified before surgery in 56% of all analyzed cases. All patients with the TLIF+ALIF approach in the GA + ESPB group had a history of chronic pain in contrast to patients with the TLIF approach in the GA group (*p* = 0.01). Lumbar spinal fusion surgery is most often performed at one (47%) or two (33%) vertebral levels. Less often, spinal fusion surgery was performed at four or five levels (4.4%).

**Table 1:** Distribution of patients in two lumbar spinal fusion surgery approaches according to the type of anesthesia.

Parameters	Total (*n* = 45)	TLIF GA (*n* = 13)	TLIF GA + ESPB (*n* = 17)	*p*-value	TLIF+ALIF GA (*n* = 11)	TLIF+ALIF GA + ESPB (*n* = 4)	*p*-value
Sex, female, *n* (%)	27 (60)	9 (69)	8 (47)	0.2	7 (64)	3 (75)	0.7
BMI, kg/m^2^	30 ± 5.4	29.2 ± 5.8	30 ± 4.7	0.7	30.2 ± 5.1	23 ± 6.1	**0.04**
ASA class, *n* (%)							
I	4 (9)	1 (7.7)	1 (6)	0.8	2 (18)	0	0.4
II	31 (69)	10 (59)	10 (77)	0.3	8 (73)	3 (75)	0.9
III	10 (22)	2 (15.4)	6 (35.3)	0.2	1 (9)	1 (25)	0.4
Comorbidities, *n* (%)							
None	13 (29)	2 (15)	4 (23.5)	0.6	5 (45.5)	2 (50)	0.9
Arterial hypertension (AH)	7 (16)	2 (12)	2 (15.4)	0.8	2 (18)	1 (25)	0.8
Diabetes mellitus (DM)	2 (4.4)	0	2 (12)	0.2	0	0	–
Rheumatoid arthritis	1 (2.2)	0	0	–	0	1 (25)	0.09
AH + DM	2 (4.4)	1 (8)	1 (6)	0.8	0	0	–
AH + Atherosclerosis + ischemic heart disease	17 (38)	7 (54)	6 (35)	0.3	4 (36)	0	0.16
AH + DM + atherosclerosis + ischemic heart disease	3 (7)	1 (8)	2 (12)	0.7	0	0	–
Chronic pain factors, *n* (%)							
Chronic pain	25 (56)	7 (54)	11 (65)	0.5	3 (27)	4 (100)	0.01
Adiposity	17 (38)	6 (46)	7 (54)	0.8	4 (36)	0	0.2
Spinal surgery in anamnesis	10 (22)	0	4 (23.5)	0.06	4 (36)	2 (50)	0.6
Anxiety	9 (20)	2 (15)	4 (23.5)	0.6	1 (9)	2 (50)	0.08
Emotional labiality	5 (11)	2 (15)	1 (6)	0.4	1 (9)	1 (25)	0.4
Sleep disorders	4 (9)	1 (8)	1 (6)	0.8	1 (9)	1 (25)	0.4
Depression	6 (13)	1 (8)	2 (12)	0.7	1 (9)	2 (50)	0.08
Surgery levels, *n* (%)							
Level 1	21 (47)	7 (54)	4 (23.5)	0.09	7 (64)	3 (75)	0.7
Level 2	15 (33)	4 (31)	8 (47)	0.4	3 (27)	0	0.2
Level 3	5 (11)	1 (7)	4 (23.5)	0.25	0	0	–
Level 4	2 (4.4)	1 (8)	0	0.2	1 (9)	0	0.5
Level 5	2 (4.4)	0	1 (6)	0.4	0	1 (25)	0.07

Data are presented as mean ± SD or number (*n*), percentage (%), and median (interquartile range). yr.: years; kg: kilogram; SAH: subarachnoid hemorrhage; CV, cerebral vasospasm; DCI, delayed cerebral ischemia; BMI, body mass index; GCS, Glasgow coma scale; ICH, intracerebral hemorrhage; ICU, intensive care unit; IQR, interquartile range; NS, not significant. *p*-value^1^ comparing GA and GA + ESPB groups undergoing TLIF surgery; *p*-value^2^ comparing GA and GA + ESPB groups undergoing TLIF + ALIF surgery.

### 3.2 Assessment of pain intensity in the first 48 hours postoperatively

**Figure 1:**
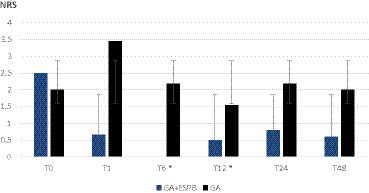
Pain score at rest between patients with general anesthesia with and without erector spinae plane block undergoing lumbar spinal fusion surgery with posterior transforaminal fusion approach. GA: general anesthesia; ESPB: erector spinae plane block; NRS: numeric rating scale; T0: before the surgery; T1: 1 h after the surgery; T6: 6 h after the surgery; T12: 12 h after the surgery; T24: 24 h after the surgery; T48: 48 h after the surgery; * – statistically significant difference.

**Figure 2:**
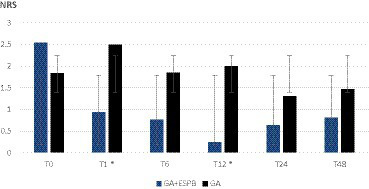
Pain score at rest between patients with general anesthesia with and without erector spinae plane block undergoing lumbar spinal fusion surgery with a combined posterior transforaminal and anterior surgical approach. GA: general anesthesia; ESPB: erector spinae plane block; NRS: numeric rating scale; T0: before the surgery; T1: 1 h after the surgery; T6: 6 h after the surgery; T12: 12 h after the surgery; T24: 24 h after the surgery; T48: 48 h after the surgery; * – statistically significant difference.

As shown in Figures 1, 2, the pain was assessed at 0, 1, 6, 12, 24, and 48 h after the surgery. We found significantly lower pain scores in GA + ESPB vs. GA patients at several time points: 6 h after the surgery, pain at rest was NRS 0 vs. 2.18 ± 1.2; *p* < 0.001 in TLIF patients. Similarly, in TLIF+ALIF surgery patients, the pain score at rest was lower already 1 h after the surgery, NRS 0.94 ± 1.3 vs. 2.5 ± 2.3; *p* = 0.04 in the GA + ESPB group compared to the GA group.

Finally, 12 h after surgery, the mean pain score at rest in the GA + ESPB vs. GA group was lower in both surgery approaches: TLIF approach was 1.54 ± 1.2 vs. 0.5 ± 066; *p* = 0.004 and in the TLIF+ALIF approach was 2 ± 1.2 vs. 0.25 ± 0.5; *p* = 0.015, respectively.

### 3.3 Opioid consumption in the first 24 hours postoperatively

In total, 73%, or 33 patients out of 45, required additional fentanyl analgesia after surgery without differences according to the type of anesthesia, as reflected in Table 2. Fentanyl immediately after surgery was less often started in those receiving GA + ESPB vs. GA alone, respectively, in 29% vs. 77% (TLIF) and in 0% vs. 82% (TLIF+ALIF); *p* = 0.01 and *p* = 0.004. Additionally, we observed that ESPB provides a good analgesic effect for up to 6.8 ± 3.2 h in the TLIF and 8.9 ± 7.6 h in the TLIF+ALIF approaches. Therefore, patients with ESPB had lower total fentanyl consumption, particularly those undergoing the TLIF+ALIF approach, 1.4 ± 0.45 mg/24 h vs. 0.9 ± 0.7 mg/24 h; *p* = 0.01, as depicted in Table 2.

**Table 2:** Distribution of opioid consumption in two lumbar spinal fusion surgery approaches according to the type of anesthesia.

Parameters	Total (*n* = 45)	TLIF GA (*n* = 13)	TLIF GA + ESPB (*n* = 17)	*p*-value	TLIF+ALIF GA (*n* = 11)	TLIF+ALIF GA + ESPB (*n* = 4)	*p*-value
Number of patients requiring fentanyl analgesia, *n* (%)							
Total, (if NRS > 6)	33 (73)	10 (77)	9 (53)	0.2	11 (100)	3 (75)	0.07
Immediately after surgery	24 (53)	10 (77)	5 (29)	0.01	9 (82)	0	0.004
Later after surgery	9 (20)	0	4 (23.5)	0.02	2 (18)	3 (75)	0.009
Time to rescue fentanyl analgesia, h	7 ± 5	1.2 ± 0	6.8 ± 3.2	0.2	1.5 ± 0.7	8.9 ± 7.6	0.15
Fentanyl consumption, mg/24 h							
Total in 24 h period	1.1 ± 0.5	0.9 ± 0.4	0.9 ± 0.6	0.99	1.4 ± 0.4	0.9 ± 0.7	0.01
Started immediately after surgery	1.2 ± 0.5	0.9 ± 0.4	1.2 ± 0.74	0.2	1.4 ± 0.5	0	0.2
Started later after surgery	0.9 ± 0.5	0	0.6 ± 0.2	0.3	1.5 ± 0.04	0.9 ± 0.7	0.02

Data are presented as mean ± SD or number (*n*) and percentage (%) and median (interquartile range) GA, general anesthesia; ESPB, erector spine plane block, h, hours; SD, standard deviation; TLIF, transpedicular fixation posterior approach; TLIF + ALIF, combined posterior and anterior approach.

## 4 Discussion

In this retrospective pilot study, we demonstrated that ESPB might be suitable for lumbar spinal fusion surgery patients with two different surgical approaches: posterior (TLIF) or combined posterior and anterior (TLIF+ALIF). We observed that ESPB reduces pain at rest in the 48 h postoperative period, providing a good analgesic effect for up to 6.8 ± 3.2 h in the TLIF and 8.9 ± 7.6 h in the TLIF+ALIF approaches. Additionally, it reduces the number of patients requiring immediate postoperative fentanyl analgesia, the initiation of the fentanyl after surgery, and the total fentanyl consumption compared to GA alone in both surgical approaches, particularly in the TLIF+ALIF approach.

Opioid requirement reduction is essential, particularly in spinal surgery patients who might be at high risk of developing chronic back pain syndrome without showing enough satisfaction after surgery. Moreover, the application of opioids is associated with major side effects such as nausea and vomiting, sedation, urinary retention, ileus, and respiratory depression. These side effects promote a longer hospital stay and a longer recovery time. In our study, 56% of patients suffered from chronic back pain (> 3 months) already before surgery (60% with TLIF and 46% with the TLIF+ALIF approach). According to other studies, for every 10% of the patients suffering from severe pain in the postoperative period, there is a 30% risk of the development of chronic pain (9, 14–22). As a result, multimodal analgesia with the application of regional blocks is gaining in popularity (23–26).

We must say that the applications of ESPB are not always allowed to fully avoid fentanyl administration for postoperative pain control. In total, 33 patients out of 45 required additional fentanyl analgesia after surgery, without differences according to the type of anesthesia. Still, ESPB allowed to reduce the total fentanyl consumption in the 24 h postoperative period with the greatest analgesic effect produced in patients with the TLIF+ALIF approach, where 81% of patients did not require fentanyl in the early postoperative period and it was started on average 8.9 ± 7.6 h after the surgery. Thereby, the total fentanyl consumption in 24 h was considerably lower in the GA + ESPB group compared to GA for those with the TLIF+ALIF approach, with MD 0.5 ± 0.23 mg/24 h; *p* = 0.015.

Interestingly, 29.4% of TLIF patients in the GA+ ESPB group required fentanyl analgesia early after the operation, but after the TLIF +ALIF approach, none of the patients required immediate fentanyl infusion. That might be explained by the small group of patients who received ESPB for TLIF+ALIF surgery.

ESPB might be useful as a part of multimodal analgesia because it also considerably reduces the initiation of fentanyl analgesia immediately after surgery. Similar results were reported by Liang et al. in a 2021 meta-analysis. They demonstrated that patients receiving ESPB in spinal fusion surgeries in the lumbar region less often required rescue analgesia (RR = 0.39, from 0.19 to 0.80, *p* = 0.01). Moreover, rescue analgesia was asked for later compared to patients without ESPB. The application of ESPB prolonged the time until rescue analgesia was started on average for 6.15 h (from 2.19 to 10.12; *p* = 0.002) (27). Our study confirmed this data, where fentanyl analgesia immediately after the surgery was more often started in the GA group vs. GA + ESPB group in both the TLIF (77% vs. 29%) and TLIF+ALIF (82% vs. 0%) approaches.

We found that the ESPB reduces total 24 h fentanyl consumption, particularly in TLIF+ALIF approach patients (MD 0.5 mg/24 h). Other studies had shown marked opioid reduction in the ESPB group patients (MD, −18.69; 95% CI, −27.95 to −9.42; *p* < 0.0001) in various spinal surgeries (20, 26–37). However, still great non-homogeneity in results regarding opioid reduction is shown. Wu et al. in 2019 found a small difference in opioid consumption in the first 24 h (MD, −2.6; 95% CI, −4.82 to −0.38; *p* < 0.0001) (27, 38). Our study showed a mean difference in fentanyl consumption of 0.26 mg. When recalculated to intravenous morphine equivalents, it is 1.3 mg. We might conclude that the different types of surgery, such as laminectomy or decompression, require a greater dose of opioids for postoperative analgesia compared with lumbar discectomy (39–44).

We also evaluated pain intensity at rest after surgery in the first 48 h postoperative period. High pain levels are usually expected after spinal fusion surgeries (4). It is reported that the peak in pain intensity after spinal surgeries in the lumbar region is usually felt 4 h after the surgery, and it gradually decreases during the next 72 h (28, 45–48). That is why we aimed to evaluate the pain level in the first 48 h after the surgery. Furthermore, there is no common opinion about when to measure pain—at rest or movement (4, 14, 49–54). We found statistically significant differences in the pain intensity between two applied types of anesthesia: 1, 6, and 12 h after surgery. In all included patients, the mean pain intensity at rest in the GA group was NRS 3, but it is important to specify that this pain level in most of the patients (87.5%) was observed when fentanyl analgesia was used. The authors describe ESPB in a wide range of spinal surgeries—decompressions, discectomies, and fusion surgeries—and in some cases, the type of surgery is not always specified (4, 11, 27, 28, 39, 55). Still, there is no clear understanding of the mechanism of action and distribution of local anesthetics after ESPB. Most likely, the analgesic effect is provided by the distribution of the local anesthetic in the dorsal and ventral nerve roots from the interfacial space between the processus transversus and the erector spine muscle group; however, systemic absorption of the local anesthetic cannot be excluded (6, 23, 35, 56–64).

The postoperative pain intensity can be affected not only by surgical trauma but also by risk factors for chronic pain (65–68). Other publications demonstrate that chronic pain before surgery increases the risk of postoperative chronic pain by 2.6 times (9, 24, 69–71). In our study, half of the patients (56%) suffered from chronic back pain (>3 months) already before surgery (60% with the TLIF approach and 46% with the TLIF+ALIF approach), but we did not evaluate the incidence of postoperative chronic pain 3 months after the surgery since the study was retrospectively designed.

The duration of the ESPB depends on the volume and concentration of the local anesthetic, as well as on the application of adjuvants (23, 54, 72–75). Rizkkalla et al., in the meta-analysis (2021) of 15 studies, showed that the duration of ESPB varied from 4 to 72 h and this was influenced by the type of local anesthetic, its volume (20–40 mL), and if the block was unilateral or bilateral (55). In our study, we unified the dose of the local anesthetic and used 30 mL of bupivacaine 0.35% bilaterally, knowing that 20 mL in the lumbar region distributes up to 2–3 levels (76) and the expected duration of the ESPB is 6–8 h after bilateral block with the 20 mL of bupivacaine 0.25% reported by Singh et al. (77). We added 200 μg epinephrin, decreasing the systemic absorption of the local anesthetic (78–81). We observed that our regimen had not affected the prolongation of the block when compared to other studies (55, 77, 82). There is no certainty about the optimal dose of the local anesthetic or volume. Studies show variable doses of the local anesthetic for spinal surgeries: 10–40 mL of ropivacaine, levobupivacaine, bupivacaine (in concentrations 0.5, 0.25%, or 0.375%), and lidocaine (in concentrations 1% or 2%), not exceeding the maximal dose (21, 67, 75).

Since the anatomical structures are being impacted during the spinal surgery, there might be different analgesic effects of ESPB, which can be affected by the changes in the anatomical structures of the spine (3, 83–85). That emphasizes the importance of the evaluation of the sensor block before and after surgery. It might be the restriction of our study that routinely ESPB was performed after induction of anesthesia without the evaluation of a sensor block. Still, we speculate that it might be hard to distinguish if the analgesic effect is always achieved by ESPB or by the systemic absorption of the local anesthetic after surgery (11, 86–88).

We admit as a major limitation of this study that it was a retrospectively designed pilot study to evaluate our first experience with ESPB. Therefore, we were not able to reach equal distributions of different TPF surgery approaches between the GA and GA + ESPB groups. The patient group in the TLIF+ALIF approach receiving GA + ESPB was too small (four patients), which may lead to a type 2 error in statistical analysis. Although we reached a statistically significant difference in 24 h fentanyl consumption in the TLIF+ALIF approach, it is still too early to draw any scientific or clinically relevant conclusions.

In contrast, we did not reach a statistically significant difference in the 24 h fentanyl consumption in the TLIF group, also indicating a too low analyzed patient sample size. Nevertheless, the data were precisely manually collected by going through each medical history, surgery, and anesthesia performed by the same surgeon and the same anesthesiologist, and postoperative care was strongly standardized for all analyzed patients. According to ASA classes and co-morbidities most patients were homogenic, although some heterogenicity was noticed in those with TLIF+ALIF approach and GA + ESPB, these patients more often presented chronic pain, anxiety and depression.

Assuming a medium effect size of 0.5, a significance level of 0.05, a power of 0.80, and a potential attrition rate of 10%, the minimum sample size required for each group would be approximately 64 participants.

## 5 Conclusion

Retrospective data from our first clinical experience with ESPB in TPF surgery patients indicate that ESPB might be an effective component of multimodal analgesia in lumbar spinal fusion surgery patients with TLIF or TLIF+ALIF surgical approaches. ESPB significantly reduced pain at rest after surgery, the number of patients requiring immediate postoperative fentanyl analgesia, and total fentanyl consumption in both surgical approaches, particularly in TLIF+ALIF. However, the application of ESPB does not always provide sufficient analgesia to completely avoid fentanyl administration after the surgery in the 24 h postoperative period. Further prospective analysis, including more patients, is necessary to confirm the effectiveness of both TPF approaches.

## Data availability statement

The raw data supporting the conclusions of this article will be made available by the authors, without undue reservation.

## Ethics statement

The studies involving humans were approved by Prof. Olafs Brūvers Theology Asoc. Prof. Santa Purviņa Pharmacology Asoc. Prof. Voldemārs Arnis Rehabilitology Prof. Regīna Kleina Pathology Prof. Guntars Pupelis Surgery Asoc. Prof. Viesturs Liguts Toxicology Doc. Iveta Jankovska orthodontology Doc. Kristaps Circenis Lecturer Ilvija Razgale. The studies were conducted in accordance with the local legislation and institutional requirements. The participants provided their written informed consent to participate in this study. Written informed consent was obtained from the individual(s) for the publication of any potentially identifiable images or data included in this article.

## Author contributions

JB: Methodology, Writing – original draft. AO: Supervision, Conceptualization, Data curation, Methodology, Writing – review & editing. LS: Conceptualization, Formal analysis, Writing – review & editing. ZG-K: Data curation, Writing – original draft. JN: Formal analysis, Writing – original draft. IL: Conceptualization, Writing – review & editing.
